# Development and validation of personalised risk prediction models for early detection and diagnosis of primary liver cancer among the English primary care population using the QResearch® database: research protocol and statistical analysis plan

**DOI:** 10.1186/s41512-022-00133-x

**Published:** 2022-10-20

**Authors:** Weiqi Liao, Peter Jepsen, Carol Coupland, Hamish Innes, Philippa C. Matthews, Cori Campbell, Eleanor Barnes, Julia Hippisley-Cox, on behalf of the DeLIVER consortium

**Affiliations:** 1 Nuffield Department of Primary Care Health Sciences, University of Oxford, Oxford, UK; 2 Department of Hepatology and Gastroenterology, Aarhus University Hospital, Aarhus, Denmark; 3 Division of Primary Care, School of Medicine, University of Nottingham, Nottingham, UK; 4 School of Health and Life Sciences, Glasgow Caledonian University, Glasgow, UK; 5 The Francis Crick Institute, London, UK; 6 University College London, London, UK; 7 Nuffield Department of Medicine, University of Oxford, Oxford, UK

**Keywords:** Liver cancer, Hepatocellular carcinoma (HCC), Cholangiocarcinoma, Risk prediction model, Early detection, Diagnosis, Symptom, Comorbidity

## Abstract

**Background and research aim:**

The incidence and mortality of liver cancer have been increasing in the UK in recent years. However, liver cancer is still under-studied. The Early Detection of Hepatocellular Liver Cancer (DeLIVER-QResearch) project aims to address the research gap and generate new knowledge to improve early detection and diagnosis of primary liver cancer from general practice and at the population level. There are three research objectives: (1) to understand the current epidemiology of primary liver cancer in England, (2) to identify and quantify the symptoms and comorbidities associated with liver cancer, and (3) to develop and validate prediction models for early detection of liver cancer suitable for implementation in clinical settings.

**Methods:**

This population-based study uses the QResearch® database (version 46) and includes adult patients aged 25–84 years old and without a diagnosis of liver cancer at the cohort entry (study period: 1 January 2008–30 June 2021). The team conducted a literature review (with additional clinical input) to inform the inclusion of variables for data extraction from the QResearch database. A wide range of statistical techniques will be used for the three research objectives, including descriptive statistics, multiple imputation for missing data, conditional logistic regression to investigate the association between the clinical features (symptoms and comorbidities) and the outcome, fractional polynomial terms to explore the non-linear relationship between continuous variables and the outcome, and Cox/competing risk regression for the prediction model. We have a specific focus on the 1-year, 5-year, and 10-year absolute risks of developing liver cancer, as risks at different time points have different clinical implications. The internal–external cross-validation approach will be used, and the discrimination and calibration of the prediction model will be evaluated.

**Discussion:**

The DeLIVER-QResearch project uses large-scale representative population-based data to address the most relevant research questions for early detection and diagnosis of primary liver cancer in England. This project has great potential to inform the national cancer strategic plan and yield substantial public and societal benefits.

**Supplementary Information:**

The online version contains supplementary material available at 10.1186/s41512-022-00133-x.

## Introduction and research background

According to the most recent statistics from Cancer Research UK, liver cancer represents the 18th most common cancer in the UK, accounting for 2% of all new cancer cases in 2017. However, it is the 8th most common cause of cancer death, accounting for 3% of all cancer deaths in 2018 [1]. Deaths due to liver cancer have increased by around 50% in the last decade [2]. The age-standardised incidence and incidence-based mortality rates of primary liver cancer increased during 1997–2017, particularly notable in hepatocellular carcinoma (HCC) [3]. The rapid increase in incidence and mortality rates is a public health concern, and a burden to health and social care. Compared with other cancers, the prognosis of liver cancer is poor, with respective 1- and 5-year survival estimates of only 38.1% and 12.7% in England during 2013–2017 [1]. Early diagnosis is associated with better survival. When it is diagnosed at its earliest stage, 78% of people can survive for 1 year or longer, compared with 20% when diagnosed at the latest stage [1, 4, 5].

There are two general approaches for early detection and diagnosis of (ED&D) cancer (World Health Organization) [6]. Early diagnosis aims to detect symptomatic patients as early as possible. For individuals *at high risk but without symptoms*, using reliable tests in screening can detect cancer at early stages. Therefore, the study design in this project is around these two approaches. A better understanding of the clinical features that indicate the development and progression of liver cancer would improve prompt referrals for investigation and intervention. Risk prediction models could estimate individual patients’ risk of developing liver cancer and identify those at high risk from the primary care population (risk stratification) for active surveillance (screening) using liver ultrasonography and serum alpha-fetoprotein (AFP) test [7]. Both approaches could help to shift the diagnosis of liver cancer towards earlier stages, when patients have a greater chance of curative treatments and better survival outcomes. All of these are the rationale and motivations to conduct the DeLIVER-QResearch project, which is part of an initiative funded by Cancer Research UK to early detect hepatocellular liver cancer (DeLIVER), project website: www.deliver.cancer.ox.ac.uk.

The QResearch team has conducted previous work on developing the QCancer prediction models to estimate future risk of lung, colorectal, gastro-oesophageal, pancreas, blood and renal tract cancers for both men and women, breast, cervical, and ovarian cancers for women, and prostate and testicular cancers for men [8]. The QCancer algorithms have been implemented in the NHS computer system as decision support tools for GPs. An online risk calculator is also available at https://www.qcancer.org/, free for public use. However, liver cancer is still not part of the QCancer model family yet. Therefore, we will develop a prediction model for liver cancer in this project.

## Research aim and objectives

This study aims to generate new knowledge for early detection and diagnosis of primary liver cancer from the English population, with a special interest in HCC. There are three research objectives in the DeLIVER-QResearch project:

1. To understand the current epidemiology of people diagnosed with liver cancer in England, including the incidence, route to diagnosis, cancer stage and histology, treatments, survival duration, and the main causes of death;
2. To explore and characterise the symptom and comorbidity profile for patients diagnosed with liver cancer, compared with those without liver cancer. The findings can help both patients and GP to achieve early diagnosis through symptomatic presentation in general practice.
3. To develop and validate personalised prediction models for estimating the risk of patients getting a diagnosis of liver cancer in the next 5 or 10 years using primary care electronic health records (EHRs), which could be used to identify patients at the highest risk who will benefit the most from active surveillance and early clinical intervention.

## Methods

### Study designs

An open cohort study will be used for research objectives 1 and 3, and a nested case–control study (within the same cohort) for research objective 2.

### Data source—the QResearch® database

Routinely collected EHRs linked to the QResearch database (version 46) will be the data source for this study. QResearch is a large consolidated database with anonymised EHRs of over 35 million patients from 1800 + general practices using the Egton Medical Information Systems (EMIS) spread across England. The database includes patients who are currently registered with the practices as well as historical patients who may have left or died. Historical records date back to 1989 with linked data on all practices since 1998. Patients’ primary care records are linked with other national datasets, such as the Hospital Episode Statistics (HES, secondary care data, including inpatient, outpatient, accident and emergency (A&E), and critical care), death registration data (up to 15 causes of death) from the Office for National Statistics (ONS), and cancer registry from Public Health England (PHE).

The team conducted a rapid literature review (including the NICE guidelines) and had clinical input from physicians (hepatology, primary care, infectious diseases) to inform the inclusion of variables and prepare code lists to extract data from the QResearch database. Two patient representatives reviewed the lists of symptoms and comorbidities and shared their experiences and disease trajectories with the lead researcher. We prepared Read/SNOMED-CT code lists to extract events from the GP records, ICD-10 code lists for diagnosed diseases in the HES, cancer registry and death records, and OPCS code lists for interventions and procedures conducted in NHS hospitals. Tables 1 and 2 summarise the variables we requested for the three research objectives. Some variables may not be significantly associated with the outcome after the analysis. However, we do not want to miss any potential association. Therefore, the variables are included as broadly and comprehensively as possible in the project set-up phase.

**Table 1:** General variables for this study

Data source	Categories	Variables
**GP record**	Demographics	Age, sex, ethnicity, socioeconomic deprivation (Townsend quintile), geographical regions in England
	Lifestyle	Body mass index (BMI, continuous variable), smoking and drinking status and intensity (with units)—longitudinal data available for all variables
**HES**		Diagnoses and treatments in the outpatient appointments and hospital admissions
**Cancer registry (PHE)**		Date of liver cancer diagnosis, route to diagnosis, stage at diagnosis, cancer grade and histology
**HES**	Treatments	Surgical resection of liver, liver transplantation, ablation of liver, transarterial chemoembolization (TACE), transarterial radioembolization (TARE)—OPCS codes
**ONS death**		Date of death, all causes of death (up to 15)

*HES* hospital episode statistics, *ONS* Office for National Statistics, *PHE* Public Health England

**Table 2:** Symptoms, comorbidities, and clinical characteristics relevant to liver cancer

Symptoms (GP records)	
Appetite and upper gastrointestinal symptoms	Change in taste, loss of taste, dry mouth/thirst, dysphagia, loss of appetite or feeling full after eating small amounts, heartburn/gastro-oesophageal reflux (GOR), indigestion, nausea, vomiting, hematemesis, stomach pain/discomfort/cramp
Abdominal and bowel symptoms	Abdominal pain (right upper quadrant), abdominal lump on the right side, abdominal distension, abdominal mass, swollen abdomen/flatulence/bloating, ascites, change in bowel habits, diarrhoea, constipation, melaena
Non-specific and systemic symptoms	Weight loss, fatigue/tiredness/lethargy (lack of energy), nausea, confusion (may suggest hepatic encephalopathy), fever, shivering, jaundice (yellow skin), pruritis (itchy skin), rash, dark urine, pale stools, night sweats, right shoulder pain, back pain, peripheral oedema, unexplained bruising
Comorbidities (GP and HES records)	
Liver diseases	Cirrhosis, hepatitis B infection, hepatitis C infection, non-alcoholic fatty liver disease (NAFLD), alcoholic liver disease, autoimmune hepatitis, liver adenoma, any other chronic liver diseases
Cardiorespiratory diseases	Hypertension, coronary heart disease, heart failure, chronic obstructive pulmonary disease (COPD), venous thromboembolism
Metabolic disorders	Type 1 and type 2 diabetes mellitus, electrolyte disorders, dyslipidaemia, obesity, gout
Other GI and systemic diseases	Chronic pancreatitis, hepatic cyst, renal cyst, pancreatic cyst, hemochromatosis, cholangitis, gallbladder stones, gastrointestinal haemorrhage, peptic ulcer disease, anaemia, thrombocytopenia
Others	Heroin use or other drug addiction, alcoholism, HIV/AIDS
Clinical and family history (GP records)	
Family history	Liver cancer, (any) gastrointestinal cancers (gastric, bowel, liver, pancreas)
Personal history	Previous cancers
Investigation and patient management (logged) in primary care records	
Blood tests	Haemoglobin, HBV (antibody/antigen/DNA), ALT, AST, PLT, BR, GGT, ESR, CRP
Medications	Furosemide, spironolactone, propranolol, carvedilol, lactulose, rifaximin, tenofovir, entecavir, lamivudine, antiretroviral therapy; statins, aspirin and metformin

*HES* hospital episode statistics, *HBV* hepatitis B virus, *HCV* hepatitis C virus, *HEV* hepatitis E virus, *ALT* alanine transaminase, *AST* aspartate aminotransferase, *PLT* platelet count, *BR* bilirubin, *GGT* gamma-glutamyl transferase, *ESR* erythrocyte sedimentation rate, *CRP* C-reactive protein

### Study setting and population

**Fig. 1:**
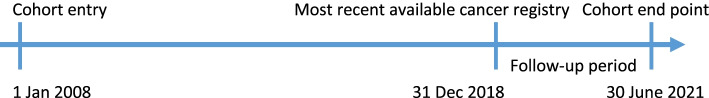
Timeline of the dynamic patient cohort for the DeLIVER-QResearch (version 46) project. Data from primary care, secondary care, and death records are available for liver cancer cases during the follow-up period, up till 30 June 2021

This is a population-based study using the QResearch database. We use similar inclusion and exclusion criteria as those in previous studies [8–10] to develop and validate the other QCancer models. The study population will be adult patients aged between 25 and 84 years old and without a diagnosis of liver cancer before the date of cohort entry. Patients diagnosed with cancer aged 16–24 years old are classified as Teenage and Young Adult (TYA) cancer [11]. The age range covers the majority of patients, who are more likely to benefit from active surveillance and screening for early diagnosis of liver cancer. The included patients need to have been registered in the general practices for at least 12 months, and these practices need to have contributed to the QResearch database for a minimum of 12 months before the cohort entry date. This is to ensure complete data before cohort entry. Figure 1 shows the timeline of the dynamic patient cohort for the DeLIVER-QResearch project.

### Identification of liver cancer cases

Incident primary liver cancer cases during 2008–2018 will be identified and followed up to 30 June 2021. The most recent available data from the cancer registry was 31 December 2018 when the data were extracted in August 2021. However, the primary care records, HES, and the death registry only had a time lag of several months. Therefore, we could know whether patients received treatments or not after diagnosis of liver cancer and the outcomes (e.g. death, left cohort, or still alive) during the follow-up period.

### Study outcomes

The primary outcome is the incident diagnosis of primary liver cancer. The date of cancer diagnosis was the earliest date recorded in the four linked data sources (primary care, hospital admission, cancer registration, and death registry), which will be the index date in the nested case–control study. The secondary outcome is the stage at diagnosis (likely to convert into a binary variable, i.e. early vs late stage).

### Control groups in the nested case–control study

Around 80–90% of patients have cirrhosis before being diagnosed with HCC [12]. Cirrhosis could be seen as one possible precursor and on the disease trajectory of developing HCC. Therefore, to better understand the clinical features and early signs of liver cancer, we will select two control groups (the general primary care population and patients diagnosed with cirrhosis) in the nested case–control study. The demographic and clinical characteristics of liver cancer cases are likely to be different from those in the general primary care population, and may differ by geographical region and over time. Therefore, up to 10 controls will be matched with each cancer case by age, sex, general practice (regions), and calendar year (time) wherever possible using incidence density sampling [13]. With such matching variables, we can better infer statistically what symptoms and comorbidities are associated with liver cancer. Each control will be allocated an index date which is the date of cancer diagnosis in their matched case. We will investigate the number of patients with and without cirrhosis in liver cancer cases. If the sample size is big enough, we will stratify liver cancer cases by cirrhosis in subgroup analysis.

### Statistical analysis plan

#### General statistical methods used in multiple research objectives

##### Descriptive statistics

Descriptive statistics will be used to describe the sociodemographic (e.g. age, sex, ethnicity, Townsend quintile) and clinical characteristics (e.g. symptoms, comorbidities, cancer stage, grade, histology) of the study population, using means and standard deviations, medians and interquartile ranges (IQR), and proportions, for different types of data as appropriate. For the nested case–control study, characteristics will be described separately for cases and the two sets of controls. We will describe the temporal changes in incidence, routes to diagnosis, stage at diagnosis and treatments for liver cancer by year during the study period.

##### Handling missing data

For symptoms, comorbidities, and medication use, the absence of information in the EHRs will be assumed that the patient did not have the health conditions or was not prescribed the medications. There may be missing data in some variables, as they were not collected and recorded in the EHRs. We will use multiple imputation with chained equations (MICE) to replace missing values for ethnicity, Townsend quintile, BMI, smoking status, alcohol intake and cancer staging with the assumption of data missing at random (MAR) [14–17]. We will first investigate the proportion of missing values in each variable. At least five imputations will be conducted, as the TRIPOD statement suggests that 5 or 10 imputations be sufficient [18]. We will conduct ten imputations if computationally feasible for the whole cohort (around 9 million patients). We can conduct more imputations in the case–control study, as the sample size is much smaller. Rubin’s rules will be used to combine the model parameter estimates across the imputed datasets [19].

#### Epidemiology of primary liver cancer (research objective 1)

The overall incidence rate of liver cancer and by age groups, sex, ethnicity, socioeconomic deprivation (Townsend quintiles), the 10 geographical regions in England, and liver cancer subtypes (HCC, cholangiocarcinoma, other specified or unspecified liver cancer) per 100,000 person-years will be calculated. Poisson regression will be used to investigate how patient characteristics are associated with the trend over time and the variation in the incidence of liver cancer and HCC throughout the study period, where interactions between age, sex, ethnicity and Townsend quintile will be explored. In addition, we will explore factors associated with emergency presentation, late stage at diagnosis, patients received curative treatments or any treatment, and survival duration, with a specific interest in the effects of sex, socioeconomic deprivation and ethnicity. Considering the importance of cirrhosis on the disease trajectory of liver cancer, we will use similar methods to describe the epidemiology (e.g. incidence, prevalence, the trend over the years, and distribution in the English primary care population) of cirrhosis using EHRs.

#### Explore the clinical features and red-flag symptoms associated with liver cancer (research objective 2)

##### The analyses for the nested case–control study

In the nested case–control study design, the cases and controls have the same length of time exposure in the risk set. The index date was the date of cancer diagnosis in cases or the equivalent date in the matched controls. In this way, it is easier to date back the exposure of symptoms (e.g. 3 months, 6 months, 1 year,… before diagnosis) and investigates the association between symptoms and the outcome at different periods to inform early diagnosis of liver cancer through symptomatic presentation. We will explore the symptoms and comorbidities in patients’ EHRs in different timeframes, e.g. up to 3 months, 4–6 months, 7–12 months, 1–2 years, 2–3 years, and 3–5 years, before the date of diagnosis in cases, or the equivalent date in controls. Multivariable conditional logistic regression will be used for the matched case–control design, adjusting for confounders (e.g. patient’s sociodemographic characteristics and lifestyle factors) and considering the interaction terms. This will determine which ‘red flag’ symptoms remain significant when other factors are taken into account in the model. After handling the missing data (subsection above), the results from the imputed datasets will be the primary estimates, but these will be compared with the estimates from two sets of sensitivity analyses, which are complete case analysis, and analyses restricted to cases and controls that have more than 3 years of EHRs before the index date. The analyses will be carried out separately for the two control groups (the general primary care population and patients diagnosed with cirrhosis), and the findings from the two control groups will be compared. After imputing cancer staging, we will conduct further analyses to identify clinical features that can help diagnose liver cancer earlier (likely to divide as binary, early vs late stages). In summary, the analyses in the nested case–control studies are more exploratory. We may develop a diagnostic prediction model in the future (depending on the findings and subjective to further funding), but not in this project. The reporting of the case–control study will follow the recommendations of the STROBE statement [20].

##### The most common symptoms and the combinations

Patients may present to their GP with a series of different symptoms. Besides each individual symptom, the frequency of the most common symptom combinations will be summarised. For individual significant symptoms and symptom combinations, we will calculate the sensitivity, specificity, and positive predictive values. Such additional information would be helpful for GPs to manage patients based on symptomatic presentation and decide whether to refer the patients to a specialist or not, especially when patients present with several non-specific symptoms.

#### Methodology for prediction models to estimate individual risk of developing liver cancer (research objective 3)

##### Sample size considerations

We used the criteria by Riley et al. [21] and the ‘pmsampsize’ package in R to calculate the minimum required sample size for developing a clinical prediction model. The parameters for sample size estimation for time-to-event outcome were set or assumed as follows. The previous QCancer prognostic models have around 30 predictors, we assume 50 predictors in the new models to allow more flexibility. The median duration from cohort entry to the incident diagnosis of liver cancer is about 10 years (QResearch has linked data on all practices since 1998, see the “Data source—the QResearch® database” section above), which is suitable for the predictive period (up to 10 years). The age-standardised incidence rate (event rate) of liver cancer in the UK during 2016–2018 was 14.5 (95% CI 14.3–14.8) per 100,000 population for men and 6.2 (95% CI 6.0–6.3) for women (statistics from Cancer Research UK [22]). A conservative ${R}_{\mathrm{Cox}-\mathrm{Snell}}^{2}$ (15% of the maximum ${R}_{\mathrm{Cox}-\mathrm{Snell}}^{2}$) was used as recommended [21]. Based on the above parameters, the minimum sample size required for developing a new model is 149,750 for men and 299,750 for women. Hence, the derivation dataset needs a minimum sample of about 450,000 men and women.

With about 9 million patients in the open cohort and an estimated 7000 incident liver cancer cases during 2008–2018 in the QResearch database, there is sufficient data for the development and validation datasets. We will use all the eligible patients in the database to maximise the power.

##### Exploration of non-linear relationships

Before imputation, a complete-case analysis will be fitted using a model containing only the continuous variables (e.g. age, BMI) within the development dataset to derive the fractional polynomial terms (up to two polynomial terms) [23, 24] for the non-linear relationships. Then a MICE model will be fitted in the development dataset to impute missing values and will include all predictors along with age interaction terms, the Nelson-Aalen estimator of the baseline cumulative hazard, and the outcome (incident liver cancer). Separate models will be fitted for men and women.

##### Model development

We will use similar established analytical strategies to develop and evaluate the risk prediction equations in this study that were used in the other QResearch studies [8, 25–29]. The internal–external cross-validation approach [30, 31] will be used to quantify the heterogeneity of predictor effects in different geographical regions in England, rather than splitting data by general practice into development and validation datasets. Eight geographical regions will be used to develop the model and the remaining two regions to validate the model. This process will be performed five times, leaving out two different regions at each time. The indicators of model performance (subsection below) from the internal–external cross-validation approach will be pooled using random-effects meta-analysis [30, 32]. The final model will be used to derive risk estimates for each year of follow-up, with a specific focus on 1-year (short-term), 5-year (medium-term) and 10-year (long-term) risk estimates. Separate models will be developed and evaluated for men and women, as the coefficients for the risk factors may be different between sexes.

Considering the application of our prediction model in clinical practice, the baseline values (evaluated at cohort entry) will be used in the model, as this is a similar clinical situation that physicians can use the current information to predict patient’s future risks when they see the patient. Cox proportional hazards model will be used as the main method to develop risk prediction models, using robust variance estimates to allow for clustering of patients within general practices, also accounting for censoring (due to death, loss of follow-up, or the end of the observation period). The proportional hazards assumptions for Cox regression will be checked. The time origin is the date of entry into the study cohort. The risk period of interest is from the time origin up to the date of incident diagnosis of liver cancer. Patients who died from other causes will be considered censored in the Cox regression. We will also consider using competing risk regression as an alternative. In such a case, death other than liver cancer before diagnosis will be competing risks. As we will use multiple imputation and fractional polynomials in such a big sample size (about 9 million patients), together with the internal–external cross-validation approach, it may not be computationally feasible to use competing risk regression. We will explore the compatibility of multiple advanced statistical techniques used together and make pragmatic decisions based on the available computing power and resources. We may use Cox regression as the main method, and draw a smaller random sample (e.g. 10%) to conduct competing risk regression as a sensitivity analysis, and compare the results between the two approaches.

The main analyses will be multivariable models including various predictors and interaction terms. The regression coefficients for each variable in the final model will be used as weights. From which, we will use a formula to derive the absolute risk estimates by combining them with the baseline survivor function evaluated for each year of follow-up, with a maximum of 10 years [33]. We will compare our developed model with the other validated risk prediction models in this field [34].

##### Variable selection and considerations

As Sauerbrei et al. pointed out [35], despite as an important topic, there is not yet enough evidence to recommend the selection of variables and functional forms in multivariable analysis. We aim to develop a parsimonious and clinically relevant prediction model. We will start with a full model by including all the variables based on clinical knowledge and research evidence from the literature. We will then remove variables that might not be clinically significant with the following criteria. We will retain *binary/categorical/ordinal variables* having hazard ratios (HR) < 0.91 or > 1.10, as HR closer to 1 may be less *clinically significant* for such variables. But the thresholds of HRs will not be used for continuous variables, as they are usually small for each incremental value. We apply a statistical significance level of 0.05 (two-tailed). To simplify the models, we will combine similar variables with comparable HRs where possible. If some variables do not have enough events to obtain point estimates and standard errors, we will combine some of these if clinically similar in nature. The variables in our prediction models are routinely recorded in the EHRs. Therefore, we can update the model periodically without burdening the healthcare professionals to collect data besides their routine work.

##### Model validation—evaluate the model performance

An imputation model (MICE) will be fitted for missing values in the validation datasets with five imputations (same as in the deviation dataset) using the methods described in the earlier subsection. We will apply the risk equations for men and women derived from the previous step to the validation data and calculate measures of model performance.

As in previous studies [36], we will calculate the *R*^2^ [37], the *D* statistic [38], the Brier score [39], Harrell’s C statistic [40] (Wolber’s C statistic [41] if using a competing risk model), and time-dependent ROC [42] at 1, 5 and 10 years and combine these across the imputed datasets using Rubin’s rules. *R*^2^ is the explained variation, where a higher value indicates a greater proportion of variation in survival time is explained by the model [37]. The *D* statistic is a measure of discrimination, which quantifies the separation in survival between patients with different levels of predicted risk, where higher values indicate better discrimination [38]. The Brier score is an aggregate measure of disagreement (the average squared error difference) between the observed and the predicted outcomes [39]. The Harrell’s *C* statistic [40] is a measure of discrimination that quantifies the extent to which those with earlier events have higher risk scores. Higher values of Harrell’s *C* indicate better performance of the model for predicting the relevant outcome. A value of 1 indicates that the model has perfect discrimination. A value of 0.5 indicates that the model discrimination is no better than chance, which is the same interpretation for time-dependent ROC. The 95% confidence intervals for the performance statistics will be calculated to allow comparisons with alternative models for the same outcome and across different subgroups [43].

We will assess the calibration of the prediction model by comparing the predicted risks at 10 years with the observed risks, and present it in a smooth calibration plot. The observed risks for men and women will be obtained by using the Kaplan–Meier estimates or cumulative incidence functions to account for competing risks. We will also evaluate these performance measures in six pre-specified age groups (25–39, 40–49, 50–59, 60–69, 70–79, 80 +) and by sex. Decision curve analysis [44] will be used to evaluate the net benefit of the prediction model (clinical usefulness). We will follow the recommendations from the TRIPOD guideline [18] to report the multivariable prognostic model.

##### Risk stratification

Risk stratification allows patients with a high predicted risk to be identified electronically from primary care records for tailored advice, active monitoring of the disease progression, and screening. Since there is no widely accepted threshold for classifying a high risk of liver cancer, we will examine the distribution of the predicted risks and calculate a series of centile values in the model. We will evaluate the sensitivity and specificity of various risk thresholds of the predicted absolute risk at the population level in the 10-year predictive horizon. We will also report how many participants with scores at least equal to each threshold, how many cancer cases will be potentially detectable, and how many cancer cases will be missed at each threshold. Such classification statistics would be useful for policymakers, if a cut-off is needed to identify patients who may benefit from surveillance and make recommendations for liver cancer screening based on risk stratification in the future. However, we will not give recommendations on which risk threshold is suitable for screening, as it needs a full cost-effectiveness analysis, which is out of the scope of the current study.

##### Dissemination and implementation plan of the prediction model

The developed algorithm will be published in peer-reviewed journals and presented at academic conferences. A web-based program can implement the new risk algorithm similar to the QCancer tool (https://www.qcancer.org/), subject to funding and the Medicines and Healthcare Products Regulatory Agency (MHRA) medical device compliance. It will also be possible to implement the risk algorithm in the EHR systems, using existing data to calculate individual risks for the primary care population. These implementation intentions will be subject to the terms and conditions of QResearch, the University of Oxford, the Cancer Research UK grant, and the agreement of all parties. It will be covered by another implementation protocol, which is out of the scope of this research protocol.

#### Summary: relevant guidelines used in this project

- NICE NG12 (Suspected cancer: recognition and referral) [45]
- The European Association for the Study of the Liver (EASL) clinical practice guidelines (management of HCC) [46]
- The STROBE statement (reporting guideline for observational studies) [20]
- The TRIPOD statement (reporting guideline for multivariable prediction model) [18, 47]

## Discussion

### The strengths and limitations of this project

To our best knowledge, this study will be the largest of its kind for early detection and diagnosis of liver cancer from the English primary care population. It has significant potential to address several important research gaps and gain a deeper understanding of the presentation, characteristics, and outcomes of liver cancer. The key strengths of this population-based study include prospective recording of predictors and outcomes, good ascertainment of outcomes through linkage of multiple national databases, and a large sample size from an established validated database (QResearch) that has been used to develop many risk prediction tools, such as QRisk3 [28], QCancer (10-year risk) [8] and other algorithms. UK primary care records have high levels of accuracy and completeness of clinical diagnoses and prescribed medications. This study has good face validity, as it is conducted in the same setting where most patients are clinically assessed, managed, and followed up in England. Thanks to the expansion and upgrade of the QResearch database in recent years, we now have information on cancer staging, grade, and histology, although there are still missing data in these variables. The rich data source makes in-depth exploration possible. This study also minimises the most common biases in epidemiological studies, such as selection bias, recall bias, and respondent bias. We will also use relevant clinical and statistical guidelines to follow the best research practice and to guide the analysis and report the findings for transparent and reproducible research. All of these strengths make the study findings more robust and more likely to generalise to the wider UK population.

The limitations of this study may include potential information bias and missing data. Based on our experiences of using primary care data, some lifestyle factors such as BMI, smoking and alcohol status may not always track the true values in real-time, and often lack consistency. In addition, the recording of family history of cancer in primary care records may be sparse. Although the completeness of cancer staging is improving year by year, we are concerned that incomplete cancer staging limits further exploration of the factors influencing early/late diagnosis of liver cancer. However, we may overcome this limitation by imputing cancer stage, as we have rich clinical data, treatments, and survival outcomes linked to the QResearch database, which can be used in the imputation model.

Due to the available resources and the funding, we will use the internal–external approach to validate the developed model using data from the same database. QResearch uses data from the EMIS, which is the computer system used by 55% of the GP surgeries in the UK. Our study population is based in England and representative of the whole English primary care population. Some previous studies [48–50] independently examined other risk equations developed by the QResearch team and concluded that using external data gave a similar performance as the internal validation approach using the QResearch database, which is reassuring. It may be possible to externally validate the developed model by our collaborators in Scotland or Denmark using datasets outside of England in future studies.

### Clinical implications and research impact

There are three key components in the DeLIVER-QResearch project: reporting the epidemiology of liver cancer in England, identifying clinical features (symptoms and comorbidities) to support earlier diagnosis of liver cancer from primary care, and developing prognostic models to identify patients at high risk but without any symptoms yet. We prioritise these research objectives as they could be linked to and contribute to the broader DeLIVER project. These studies will generate new knowledge that will allow us to gain a deeper understanding of the current situation of liver cancer in England, and inform health policy on early detection, diagnosis, and management of liver cancer at the population level. Individuals at the highest risk of developing liver cancer are most likely to benefit from active surveillance using liver ultrasonography and serum alpha-fetoprotein test. Applying personalised risk prediction models to select individuals at high risk from the population for screening could be a cost-effective approach to improve early diagnosis of liver cancer and patient outcomes, without unduly burdening the overstretched NHS, and avoiding harm to patients at low risk. The implementation of the prediction model will allow the public to calculate individual risks of developing liver cancer for free, like the other QCancer tools, which is a way to engage the public to be more aware of their health status and initiate help-seeking when necessary. The UK government and NHS England have committed to improving early diagnosis and cancer outcomes [51]. This project has great potential of making contributions to the national early detection and diagnosis cancer strategic plan, with associated public and societal benefits, such as reducing premature death due to liver cancer, reducing care costs for the NHS, and reducing cancer burden to the society.

## Supplementary Information

**Additional file 1.** Co-investigators and members in the DeLIVER consortium.

## Data Availability

Due to the sensitive nature of anonymised patient-level health records and the agreement with the data provider, the data are only accessible to the named researchers of the team, stored and analysed in a data safe haven (the QResearch server).
